# Leaf energy balance modelling as a tool to infer habitat preference in the early angiosperms

**DOI:** 10.1098/rspb.2014.3052

**Published:** 2015-03-22

**Authors:** Alexandra P. Lee, Garland Upchurch, Erik H. Murchie, Barry H. Lomax

**Affiliations:** 1 The School of Biosciences, University of Nottingham, Sutton Bonington Campus, Sutton Bonington, Leicestershire LE12 5RD, UK; 2 Department of Biology, Texas State University, 601 University Drive, San Marcos, TX 78666, USA

**Keywords:** basal angiosperms, evolution, modelling, leaf size, thermal tolerance, ancestral ecology

## Abstract

Despite more than a century of research, some key aspects of habitat preference and ecology of the earliest angiosperms remain poorly constrained. Proposed growth ecology has varied from opportunistic weedy species growing in full sun to slow-growing species limited to the shaded understorey of gymnosperm forests. Evidence suggests that the earliest angiosperms possessed low transpiration rates: gas exchange rates for extant basal angiosperms are low, as are the reconstructed gas exchange rates for the oldest known angiosperm leaf fossils. Leaves with low transpirational capacity are vulnerable to overheating in full sun, favouring the hypothesis that early angiosperms were limited to the shaded understorey. Here, modelled leaf temperatures are used to examine the thermal tolerance of some of the earliest angiosperms. Our results indicate that small leaf size could have mitigated the low transpirational cooling capacity of many early angiosperms, enabling many species to survive in full sun. We propose that during the earliest phases of the angiosperm leaf record, angiosperms may not have been limited to the understorey, and that some species were able to compete with ferns and gymnosperms in both shaded and sunny habitats, especially in the absence of competition from more rapidly growing and transpiring advanced lineages of angiosperms.

## 1. Introduction

The inferred ecological preferences of the earliest angiosperms have varied extensively over the last century. Traditionally, extant woody angiosperms assigned to the paraphyletic grade known as magnoliids were considered the model for primitive angiosperm ecology, based on their possession of numerous postulated primitive features such as large bisexual flowers, free and undifferentiated perianth parts, monosulcate pollen, putatively primitive wood and numerous stamens and carpels. In particular, the order Magnoliales and the vesselless family Winteraceae were thought to model early angiosperm ecology as large-leaved, slow-growing trees or shrubs of the understorey of tropical forests [1–4]. This ecology was consistent with the proposed diversification of flowering plants in tropical uplands during the Triassic and Jurassic, and their entry into lowland environments during the mid-Cretaceous [5,6].

Re-examination of the early angiosperm record of the mid-Cretaceous Potomac Group and other units by Doyle and Hickey provided an alternative view [7–9]. The oldest angiosperm leaf macrofossils from the Potomac Group (palynozone Zone I) are rare, of low diversity, often small-leaved and restricted to stream margin environments, indicative of frequent disturbance. By contrast, younger Potomac Group angiosperms (middle Albian to early Cenomanian palynozones II and III) can be locally dominant, are of higher diversity, are larger-leaved, and occur in both stream margin environments and floodplain environments indicative of lower disturbance [7,8]. Physiognomic analysis of Potomac Group and other Cretaceous angiosperm leaves indicates a major diversification in light-gathering strategies during the mid-Cretaceous, and the appearance of diverse leaf types characteristic of trees during the later Albian and Cenomanian [7,8,10].

Subsequent molecular systematic and ecophysiological analysis of extant angiosperms, and the discovery of new Early Cretaceous angiosperm localities from outside of North America, have led to refinements and/or new hypotheses. The ‘dark and disturbed hypothesis' [11] proposes that the first angiosperms were plants of deep shade adapted to disturbed conditions. Basal living angiosperms, belonging to the orders Amborellales and Austrobaileyales, and the family Chloranthaceae, are predominantly slow-growing plants of the shaded understorey of tropical to subtropical forests, with anatomical and physiological adaptations to growth under low-light conditions [11–13]. Parsimony analysis of extant angiosperms indicates that growth under shaded conditions is the ancestral state, with the aquatic Nymphaeales and certain members of Austrobaileyales and Chloranthaceae representing ‘breakout groups' that grow in full sun. The rapid growth that characterizes most extant angiosperms does not evolve until higher in the angiosperm phylogeny, in the lineages representing magnoliids, eudicots and monocots [11–13].

Alternatively, the ‘wet and wild hypothesis' [14] proposes that growth under aquatic conditions was common among the oldest angiosperms [15]. The oldest angiosperm leaf localities in Europe and Asia are lacustrine beds dated as Barremian to Aptian (*ca* 129–113 Ma). Many of the known angiosperm taxa show obvious adaptations to the aquatic habit, such as finely dissected leaves, while those with terrestrial leaf morphology show evidence for growth along the margins of freshwater [15,16]. Intriguingly, the most widely discussed early fossil angiosperm *Archaefructus* [17], and *Hyrcantha* [18], also fit into the aquatic hypothesis. In Europe, the first appearance of angiosperm megafossils in non-lacustrine floodplain environments is during the Albian as plants of the shaded understorey. This appearance has been associated with geological evidence for loss of aridity, loss of open-canopy vegetation, increased rainfall and the origin of closed canopy forests [16].

Re-examination of the Potomac Group provides a mixed signal regarding the habitat preferences of early angiosperms, and in particular, their preference for sun or shade. Zone I angiosperm leaves belonging to basal lineages, and later Cretaceous relatives of extant basal angiosperms, have the low vein density and/or low stomatal pore area indicative of low gas-exchange rates [19], as predicted by the dark and disturbed hypothesis [11]. Low vein density is a uniform feature of all Zone I leaves from the Potomac Group, and characterizes not only taxa belonging to basal lineages but also taxa related to eudicots and magnoliids, which today are characterized by higher vein densities than ferns, gymnosperms and basal angiosperm lineages [12,20,21]. This implies that low vein density, and by extension preference for shaded habitats, was a feature of most or all early angiosperms, and that it was lost by many lineages later in the Cretaceous [19].

Contrasting with evidence from vein density are the small leaf size, sedimentology and floristic composition of some of the oldest angiosperm leaf assemblages, which appear to indicate a wider range of ecology than predicted by the dark and disturbed hypothesis. Many Zone I angiosperm leaves are ecological microphylls (225–2025 mm^2^) [10], reducing the interception of solar radiation. This, combined with a relatively greater rate of convective heat exchange, results in small leaves being more closely coupled to air temperature and therefore cooler than their larger counterparts (e.g. [22,23]). Thus, small-leaved early angiosperms may have been able to survive in fully exposed conditions. Wider analysis of some early angiosperm leaf assemblages such as Drewrys Bluff shows evidence for angiosperm growth under fully illuminated conditions, based on the dominance in the macroflora of herbaceous to shrubby angiosperms and Gnetales, and the absence of obvious trees such as conifers [24,25]. This contrasts with coeval early angiosperm leaf assemblages such as Fredericksburg, which show evidence for angiosperm growth in shaded understorey conditions based on large leaf size and occurrence in high-diversity macrofossil assemblages with abundant and diverse conifers, cycadophytes and ferns [7,8,20,26].

To address apparent conflicts in the early angiosperm record we use a leaf energy balance model to predict leaf temperature and evaluate proposed ecological roles of early angiosperms. This approach has been used to examine the evolutionary origin of terrestrial planate leaves [27,28] and potential size-dependent high temperature injury to leaves across the Triassic–Jurassic boundary [23]. From these temperature predictions it is possible to evaluate competing hypotheses by determining the critical leaf size below which an early angiosperm leaf could be fully illuminated and yet avoid lethal temperatures. Establishing whether small leaf size could compensate for low transpirational cooling capacity under fully illuminated conditions may prove key in placing the early angiosperms in an ecophysiological framework.

## 2. Material and methods

### (a) Model description

Through use of equation (2.1) [29, eq. (14.6)], the temperature of fossil leaves (T_*l*_) can be modelled as a function of air temperature (T_*a*_), radiation load, leaf area, wind speed, relative humidity (RH) and stomatal conductance (*gs*),2.1
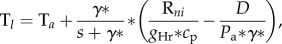
where T_*l*_ is leaf temperature (°C), T_*a*_ is air temperature (°C), γ* is apparent psychometer constant (C^−1^), *s* is slope saturation mole fraction function (Δ Pa^−1^), R_*ni*_ is isothermal net radiation (W m^−2^), *g*_Hr_ is sum of boundary layer and radiative conductances (mol m^−2^ s^−1^), *c*_p_ is specific heat of air at constant pressure (J mol^−1^ C^−1^), *D* is vapour deficit of air (kPa) and *P*_a_ is atmospheric pressure (kPa).

γ*, *c*_p_ and *P*_a_ are constants, while *s*, R_*ni*_, *g*_Hr_ and *D* are temperature dependent (this dependency is accounted for in all simulations). The net radiation absorbed by a leaf can vary as a function of many factors that affect leaf surface temperature; therefore, solar radiation was represented in the model as isothermal net radiation (R_*ni*_), which is preferred in predictive studies as it is independent of surface leaf temperature. A marginal adjustment must be made to R_*ni*_ when air temperature is changed; this is approximately equivalent to a reduction of 2 W m^−2^ per degree decrease. R_*ni*_ at the highest air temperature modelled in this study, 39°C, was set at 300 W m^−2^ for full irradiance and lowered to 50 W m^−2^ to model shade [29]. Fossil *gs* was taken from [20], who quantified the relationship between leaf vein density and gas-exchange rates in basal extant angiosperms and inferred this relationship onto fossil material to gain estimates of *gs* termed *gs*^*vein*^. Modern species commonly show a reduction in *gs* when exposed to elevated CO_2_, which maximizes water use efficiency (WUE) while avoiding a reduction in CO_2_ assimilation rates. To account for the elevated CO_2_ concentrations of the early Cretaceous (1500–2000 ppm [30]), T_*l*_ was modelled at both *gs*^*vein*^ and *gs*^*vein−25%*^. Furthermore, as the model is not entirely comprehensive of all the variables that could influence *gs*, such as how photosynthesis feeds-back to affect stomatal conductance [31], T_*l*_ was also modelled with *gs*^*vein+25%*^. This demonstrates a full span of leaf temperature sensitivity to potential fluctuations and over/underestimates of fossil *gs*.

The air temperature at which leaf temperature stress begins is species specific, but for the purpose of this model the threshold for temperature stress was set as 40°C. This is a highly conserved heat limit for CO_2_ uptake across a wide range of extant taxa [32]. It is worth noting that early Cretaceous elevated atmospheric CO_2_ levels could possibly have mitigated some of the effects of temperature stress on early angiosperm leaves. With increasing temperatures, CO_2_ availability becomes increasingly limiting to photosynthesis as the increase in carboxylase activity is typically offset by increased rates of oxygenation of Rubisco, resulting from a reduction in the solubility of CO_2_ and lowered CO_2_ affinity. Increased atmospheric CO_2_ alone would reduce this offset, possibly enabling plants to maintain photosynthetic rates at higher temperatures, as is typically seen for leaves of C_3_ plants [33]. However, there is limited knowledge on adaptation of metabolism to atmospheric CO_2_ [34], so, for the purpose of this model, stress was set at 40°C, which is a reasonable estimate for current C_3_ species.

### (b) Model experiments

#### (i) Sensitivity analysis and validation

Sensitivity analysis was performed to assess the relative weight environmental variables, T_*a*_, R_*ni*_, wind speed and RH, had on determining T_*l*_ in relation to leaf area. Analysis was run on the fossil species *Quercophyllum tenuinerve* and *Ficophyllum crassinerve,* the smallest and largest leaves respectively. To separate the relative effects of T_*a*_ and R_*ni*_, the sensitivity analysis demonstrated the effect of varying wind speed and RH on leaf temperature. In subsequent model simulations, wind speed was fixed at 1.5 m s^−1^ and relative humidity at 70% in the understory and 60% in the open, simulating a light breeze in a humid Cretaceous environment. A high humidity was chosen because of multiple lines of evidence for high humidity in the middle Cretaceous Dakota Formation, including epiphyllous mosses and leaves with tropical rainforest physiognomy [35,36]. Model validation was achieved by comparing measured T_*l*_ of *Laurus nobilis* with modelled solutions. Data indicate the model predicts both leaf temperature (*p* ≤ 0.001, *R*² = 0.83) and the leaf to air temperature difference (*p* ≤ 0.001, *R*² = 0.71) with a high degree of statistical certainty (see electronic supplementary material for full details of validation).

#### (ii) Simulation I

The relationship between leaf area and leaf temperature was modelled at three air temperatures (37°C, 38°C and 39°C) to determine critical constraints on leaf size at varying air temperatures. These runs were made on hypothetical leaves with stomatal conductance set at 0.169 mol H_2_O m^−2^ s^−1^, the maximal *gs* estimated for the common ancestor of extant angiosperms [37]. Simulations were repeated with *gs* set at 0.01 mol H_2_O m^−2^ s^−1^ to establish at what size early angiosperm leaves could avoid temperature stress at midday, when stomatal closure would be likely to occur.

#### (iii) Simulation II

The second simulation used stomatal conductance estimated from vein density (*gs*^*vein*^) [20] and leaf area data for 14 extinct early angiosperm species to model leaf temperature at T_*a*_ 39°C in both the sun and shade. This temperature was chosen as a maximum estimate of Early Cretaceous summer temperature at the fossil palaeolatitude (25–35° N) [38]. This was also highlighted by the sensitivity analysis as the critical temperature before even the smallest leaves began modelling stress. As in simulation I, T_*l*_ was also modelled with full stomatal closure.

#### (iv) Simulation III

The third simulation was set with the same environmental inputs as simulation II but with air temperature lowered to 37°C as a more conservative estimate of Cretaceous temperature. Only sun simulations were run as no species modelled temperature stress in the shade at 39°C. Sensitivity analysis suggested that this would be the highest temperature where only the very largest fossil leaves model temperature stress.

#### (v) Leaf fossils

Multiple T_*l*_ estimates were modelled for 14 species of early angiosperm leaves analysed by Feild *et al.* [19]. These include eight species from the lower part of the Potomac Group (Zone I, or Aptian to lower Albian) and six species from the Dakota Formation of Kansas and Nebraska that represent probable relatives of extant basal angiosperms (equivalent of Potomac Group palynosubzones IIC and Zone III, or uppermost Albian to Cenomanian; see electronic supplementary material for full details). The Potomac Group leaves represent much of the morphological variation from Zone I and include relatives of extant lineages that live in the shaded understorey and possible relatives of sun-tolerant ‘breakout’ taxa [11]. Possible ANA grade taxa include *Proteaephyllum reniforme*, a possible early relative of Nymphaeales, and *F. crassinerve*, which has leaves with features similar to Austrobaileyaceae [8,19,21,39]. Leaves with features of leaf architecture and opposite phyllotaxy indicative of a relationship to extant Chloranthaceae include *Moutonia* sp. (Drewrys Bluff Leaf Type 1) of Upchurch [21], *Q. tenuinerve* and *Celastrophyllum* sp. of Upchurch [21,24]. Other analysed Zone I taxa have more poorly understood affinities and include *Eucalyptophyllum oblongifolium*, which may relate to Austrobaileyales or Chloranthales, and *Rogersia angustifolia*, which has a possible relationship to Canellales. The Dakota Formation leaves analysed in this study represent probable relatives of basal lineages and Chloranthales. Probable basal lineages include *Longstrethia varidentata*, which shows a mosaic of venational and cuticular features found in the families Trimeniaceae and Schisandraceae (Austrobaileyales), and the related *Longstrethia aspera* [19,40,41]. Possible relatives of Chloranthaceae include *Crassidenticulum decurrens, Crassidenticulum landisae, Densinervum kaulii* and *Reynoldsiophyllum nebrascense* [20,21].

Fossil species were primarily selected based on age and also on the availability of leaf area and stomatal conductance data in the form of *gs*^*vein*^ [20]. Fossil leaves from Zone I of the Potomac are seldom found complete and are relatively rare components of the overall flora, which limits the sample size available for leaf area measurements. For each angiosperm species, leaf area data were taken from figured examples in the literature [8,24,40–43]. This methodological approach limits within-species replication as generally only one leaf was figured per species. However, examination of the available specimens indicates that leaf size derived from published illustrations is representative. The use of figured specimens has also been used in previous studies to determine leaf energy balance [28]. Despite these limitations, this study represents an important step in quantifying leaf temperature of extinct early fossil angiosperms, allowing for a full ecophysiological evaluation of competing hypotheses on the ecological preference of early angiosperms.

## 3. Results

### (a) Sensitivity analysis

**Figure 1.**
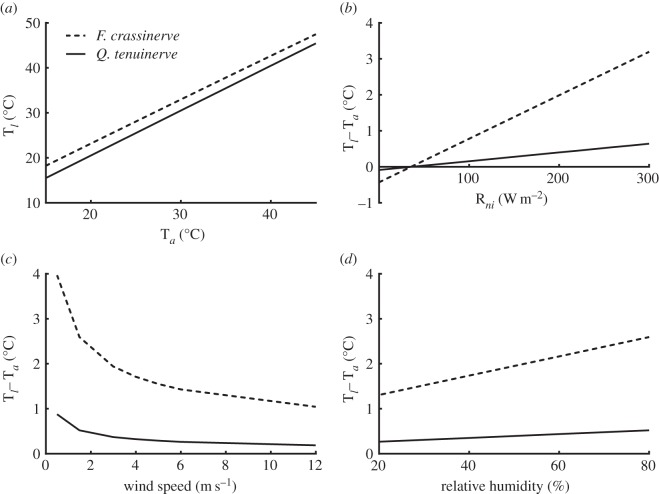
The modelled effect of varying parameters: (*a*) T_*a*_, (*b*) R_*ni*_, (*c*) wind speed and (*d*) RH on leaf temperature for the largest (*F. crassinerve*) and smallest (*Q. tenuinerve*) Cretaceous-aged fossil leaf species.

Model variables had a greater effect on the largest-leaved species *F. crassinerve* compared with the smaller-leaved *Q. tenuinerve*. With increasing T_*a*_ from 15°C to 45°C, the smallest leaf, *Q. tenuinerve,* consistently modelled T_*l*_ less than 0.5°C above air temperature. However, the largest leaf, *F. crassinerve,* modelled a minimum of 2.4°C above T_*a*_ (figure 1*a*). Under increasing R_*ni*_ (solar radiation) from 0 W m^−2^ (darkness) to 300 W m^−2^ (full sun) with T_*a*_ fixed at 39°C, T_*l*_ of *F. crassinerve* rose by over 3°C, while T_*l*_ of *Q. tenuinerve* rose by only 0.6°C (figure 1*b*). Increasing wind speed from 1.5 m s^−1^ (light) to 12 m s^−1^ (strong) reduces the T_*l*_ of *F. crassinerve* by 3°C. Comparatively, the same increase in wind speed has a relatively small effect on *Q. tenuinerve* and reduces T_*l*_ by 0.7°C (figure 1*c*). A modelled increase in RH from 20% to 80% increases T_*l*_ of *F. crassinerve* by 1.7°C. However, the same increase raises T_*l*_ of *Q. tenuinerve* by 0.3°C (figure 1*d*).

### (b) Simulation I

**Figure 2.**
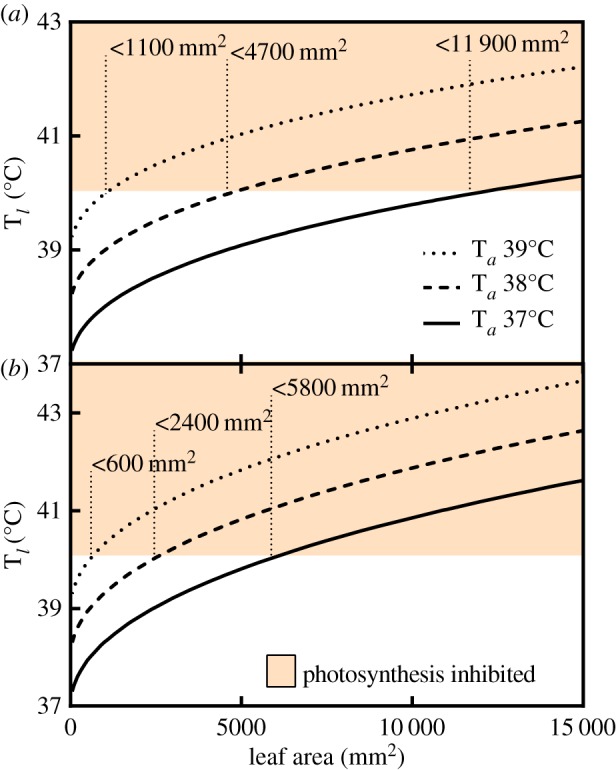
(*a*) The modelled relationship between leaf temperature and leaf area at three air temperatures with stomatal conductance set at 0.169 mol H_2_O m^−2^ s^−1^, the maximal *gs* estimated for the common ancestor of extant angiosperms [34] and (*b*) with stomatal closure. (Online version in colour.)

At 39°C, 38°C and 37°C leaves with areas less than 1100, 4700 and 11 900 mm^2^, respectively, remain within viable photosynthetic temperatures (figure 2*a*). However, with full stomatal closure at midday, critical leaf size is reduced to 600, 2400 and 5800 mm^2^ at 39°C, 38°C and 37°C, respectively (figure 2*b*).

### (c) Simulations II and III

**Figure 3.**
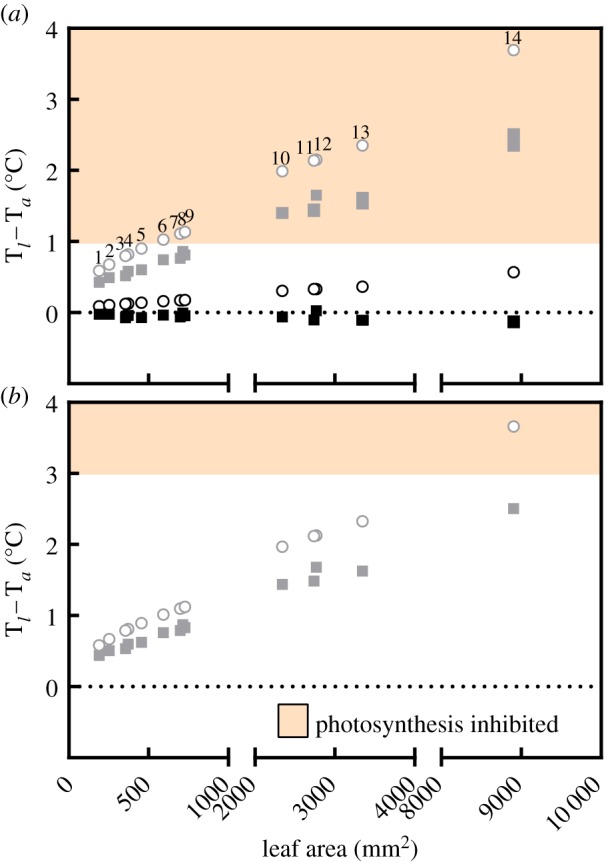
Modelled leaf temperatures for 14 early angiosperm species plotted by leaf area at 39°C (*a*) and 37°C (*b*). Open circles represent T_*l*_ − T_*a*_ with full stomatal closure while solid coloured rectangles represent the range of T_*l*_ − T_*a*_ modelled for each species with *gs*^*vein±25%*^. Black rectangles represent shade and grey rectangles sun. Numbers refer to fossil species in order of increasing leaf area: 1, *Q. tenuinerve*; 2, *Celastrophyllum* sp. from Drewry's Bluff; 3, *L. varidentata*; 4, *E. oblongifolium*; 5, *D. kaulii*; 6, *Moutonia* sp. (Drewry's Bluff Leaf Type #1); 7, *R. angustifolia*; 8, *P. reniforme*; 9, *C. landisae*; 10, *V. multifidum*; 11, *L. aspera*; 12, *R. nebrascense*; 13, *C. decurrens*; 14, *F. crassinerve*. (Online version in colour.)

At 39°C (figure 3*a*), 9 of the 14 early angiosperm species narrowly model within viable photosynthetic temperatures in full sun even with the minimum level of transpirational cooling allowed by *gs*^*vein–25%*^. However, three of these nine rise into photosynthetic inhibitory temperatures with stomatal closure. With R_*ni*_ reduced to 50 W m^−2^ to represent shade, no species models temperature stress. In simulation III, with temperature lowered to 37°C (figure 3*b*), no species models temperature stress with *gs*^*vein±25%*^ and only the largest fossil species from this study—*F. crassinerve*—models stress with full stomatal closure.

## 4. Discussion

Sensitivity analysis indicates that environmental factors have a far greater impact on leaf temperature with increasing leaf size, indicating that habitat preference for sun or shade would be more critical in larger leaved species. Modelled fossil leaf temperature indicates that the small size of many early angiosperm leaves could have compensated for their low transpirational cooling capacity and enabled survival in full sun. These results, while not invalidating the dark and disturbed hypothesis, are consistent with evidence for early angiosperms possessing a range of ecological diversity that included plants of both shaded and open habitats.

### (a) Understorey

When T_*l*_ is modelled for a shaded leaf with T_*a*_ set at 39°C, all fossil species model leaf temperatures within viable photosynthetic limits. However, in full sun at 39°C five species modelled significant temperature stress, suggesting that some early angiosperms were limited to shaded environments. The most convincingly understorey species of our dataset—*F. crassinerve*—is also the most strikingly different from most other early angiosperms. *F. crassinerve* has uncharacteristically large leaves (8906 mm^2^) compared with the majority of other early angiosperm species in our dataset (mean 1750 mm^2^ ± 2250 mm^2^) and occurs at the fern- and gymnosperm-rich Fredericksburg locality of the Potomac Group [8]. A modelled solution of leaf temperature indicates that *F. crassinerve* would have overheated in full sun at midday even at the lower 37°C estimate of Cretaceous summer temperature. This indicates that at least some Aptian–early Albian angiosperms were restricted to the shaded understorey, as predicted by the dark and disturbed hypothesis. It has been proposed that *Ficophyllum* represented an early invasion of the understorey environment by primitive angiosperms [8], based in part on a proposed relationship of *Ficophyllum* to Magnoliales in the paraphyletic (pre-APG) sense (e.g. [4]). Thus, our leaf energy balance calculations, which suggest that *Ficophyllum* was restricted to the understorey, are consistent with the dark and disturbed hypothesis from both a phylogenetic and ecophysiological standpoint.

### (b) Open, exposed

All species from the early Albian Drewrys Bluff locality (*Celastrophyllum*, *Moutonia* sp. and *E. oblongifolium*) fall below the critical leaf size to remain at photosynthetically active temperatures in full sun at 39°C. All three species have leaf area less than 600 mm^2^ and avoid overheating even with full stomatal closure. Species diversity at Drewrys Bluff is lower than Fredericksburg, and there is an absence of any obvious trees in the macroflora. Therefore, the Drewrys Bluff locality could feasibly represent an open exposed sunny environment inhabited by early angiosperms.

The Federal Hill locality in the Potomac Group (upper zone I, Albian), contained only one angiosperm leaf specimen suitable for this study—*Vitiphyllum multifidum.* The leaf area of *V. multifidum* (approx. 2300 mm^2^) places it in the mid-range of early angiosperm leaf sizes, which model heat stress at 39°C but not at 37°C. Leaf traits such as irregular venation and lack of bracing by veins have led to the suggestion that *V. multifidum* may be semi-aquatic [8]. If *V. multifidum* is indeed semi-aquatic/aquatic, this could present an adaptive mechanism for growing in full sun at higher temperatures without overheating.

Modelled leaf temperatures indicate that many leaves with chloranthoid features were potentially capable of surviving in full sunlight. According to Feild *et al.* [11], two lineages of extant Chloranthaceae represent breakout groups that have adapted to growth under full sunlight: *Hedyosmum* and *Ascarina* (minus *A. solmsiana*, which is sister to the remaining species of the genus). The fossil chloranthoid leaves examined here cannot be placed within any extant genus of Chloranthaceae, but have features suggestive of a relationship to the family. These include pinnate venation, chloranthoid teeth with thickened tissue at the apex, festooned or simple craspedodromous secondary venation, and exmedially oriented, reticulate higher-order venation, similar to what occurs in extant *Ascarina*. Other features favouring a relationship to Chloranthaceae include laminar secretory bodies, inferred to be the remains of oil cells, and opposite phyllotaxy [21]. Our model indicates that some of these leaves were capable of surviving in full sunlight, like many extant members of Chloranthaceae.

*Longstrethia varidentata* and *L. aspera* show somewhat different responses to full sunlight, with *L. varidentata* barely able to maintain photosynthetically viable temperatures under full stomatal closure. The genus *Longstrethia* has a mosaic of venational and cuticular features found in the families Schisandraceae and Trimeniaceae, currently placed within the order Austrobaileyales [19,40]. According to the analyses of Feild *et al.* [11], the ability to grow in full sun evolved five times within the two families. *L. varidentata* occurs in latest Albian strata from the Rose Creek locality of the Dakota Formation [40], where the majority of angiosperm species have the small leaf size consistent with fully illuminated conditions (cf. [44]).

Without a combined phylogenetic analysis of extant basal angiosperms and early fossil angiosperm leaves, it is not possible to conclusively determine whether modelled fossil leaf temperatures contradict the dark and disturbed hypothesis or whether these fossil species simply represent extinct relatives of proposed breakout groups. However, the uniformly low vein densities in all Zone I angiosperms and the consistency of modelled leaf temperatures for a range of small-leaved taxa suggest to us that a number of early angiosperms probably grew under well-illuminated conditions, and were able to overcome the constraints imposed by low transpiration rates through the production of small leaves.

Extant basal angiosperms (in particular, non-aquatic taxa with low gas-exchange rates) are presently restricted to their current habitats by over 100 million years of competition, with more derived angiosperm lineages characterized by significantly higher vein densities and photosynthetic rates [12,20]. In an Early Cretaceous world without the constraint of rapidly growing and rapidly transpiring angiosperms, slow-growing basal angiosperms might have successfully competed with gymnosperms and ferns in both shaded and sunny habitats as a function of their more rapid and more energetically efficient life cycle (e.g. [45]), increased hydraulic efficiency and potential for greater diversity of life form (cf. [46]). We propose that, during the earliest phases of angiosperm evolution, small-leaved and slow-growing angiosperms successfully competed with slow-growing ferns and gymnosperms in both shaded and sunny habitats, only to be later outcompeted in sunny habitats by more derived angiosperms with the rapid photosynthetic and growth rates and large stature characteristic of modern trees. We suggest that future studies test this hypothesis through the detailed analysis of functional morphology, paleoecology and phylogeny in early Albian and older angiosperm leaf fossils from the Potomac Group and elsewhere.
